# Susceptibility Testing of Fungi to Antifungal Drugs

**DOI:** 10.3390/jof4030110

**Published:** 2018-09-15

**Authors:** Maurizio Sanguinetti, Brunella Posteraro

**Affiliations:** 1 Institute of Microbiology, Fondazione Policlinico Universitario A. Gemelli, Istituto di Ricovero e Cura a Carattere Scientifico (IRCCS), Università Cattolica del Sacro Cuore, 00168 Rome, Italy; 2 Institute of Medical Pathology and Semeiotics, Fondazione Policlinico Universitario A. Gemelli, IRCCS, Università Cattolica del Sacro Cuore, 00168 Rome, Italy; brunella.posteraro@unicatt.it

**Keywords:** antifungal susceptibility, *Candida*, *Aspergillus*, CLSI, EUCAST, MALDI-TOF MS

## Abstract

Susceptibility testing of fungi against antifungal drugs commonly used for therapy is a key component of the care of patients with invasive fungal infections. Antifungal susceptibility testing (AFST) has progressed in recent decades to finally become standardized and available as both Clinical and Laboratory Standards Institute (CLSI) and European Committee on Antimicrobial Susceptibility Testing (EUCAST) reference methods and in commercial manual/automated phenotypic methods. In clinical practice, the Sensititre YeastOne and Etest methods are widely used for AFST, particularly for sterile site isolates of *Candida*. Nevertheless, AFST is moving toward new phenotypic methods, such as matrix-assisted laser desorption ionization time-of-flight mass spectrometry (MALDI-TOF MS), that are capable of providing rapid, and potentially more actionable, results for the treating clinician. Our objective is to summarize updated data on phenotypic methods for AFST of *Candida* and *Aspergillus* species and to assess their significance in view of opposing, but emerging, molecular genotypic methods.

## 1. Introduction

Invasive fungal infections, especially those caused by the species *Candida* and *Aspergillus*, continue to rise in frequency [1] and, alarmingly, are associated with antifungal resistance [2], which makes the management of patients with such infections particularly challenging [3,4]. Except for infections due to inherently antifungal-resistant species, the majority of these infections are clinically treatable by three currently available antifungal drug classes: triazoles (fluconazole, itraconazole, voriconazole, posaconazole, isavuconazole), echinocandins (anidulafungin, caspofungin, micafungin), and polyenes (amphotericin B-deoxycholate with its lipid and liposomal formulations) [5,6]. Paralleling the relatively recent introduction of new antifungal drug classes [7] and the discovery of novel agents [8] for the treatment of invasive fungal infections, the field of antifungal susceptibility testing (AFST) has progressed tremendously in the past several years [9]. Apart from practical, reliable, and reproducible laboratory methods for AFST, there has been a considerable push toward methods able to correlate in vitro laboratory tests with clinical outcome and identify new, clinically important resistance mechanisms, as has been done for susceptibility testing of bacteria [10].

The goal of performing AFST is to produce actionable data for the treating clinician on the susceptibility, intermediate (or dose-dependent) susceptibility, or resistance phenotype for an organism–antifungal agent combination. In a viewpoint article published ~15 years ago, Rex and Pfaller [11], while discussing the accuracy of the “90–60 rule” by which AFST can predict the outcome of treatment, said that AFST “has indeed come of age as a useful clinical tool.” This ultimately came true later through not only refinement of the Clinical and Laboratory Standards Institute (CLSI) and European Committee on Antimicrobial Susceptibility Testing (EUCAST) reference methods currently in place [12,13,14,15], but also expansion of commercial and automated methods for AFST [16].

All of these achievements increased the likelihood that testing several organism–drug combinations (most notably *Candida* species and the azole antifungal agents) could usefully influence the selection of therapy, thus aiding clinicians in the management of difficult-to-treat fungal infections [17]. Meanwhile, better consistency and accuracy of testing, along with clinical outcomes and pharmacokinetic/pharmacodynamic data, led to the creation of well-validated clinical breakpoints (CBPs), at least for azoles and common *Candida* species [9]. However, in lieu of CBPs, establishing epidemiological cutoff values (ECVs) helped to distinguish wild-type (WT) isolates from those that may harbor an acquired resistance mechanism and are less likely to respond to a given antifungal agent (non-WT) [18].

The objectives of this paper are to provide updates on new data from AFST studies and to discuss how AFST might improve outcomes of invasive fungal infections. In view of their clinical importance, we will focus on AFST of *Candida* and *Aspergillus* species.

## 2. Conventional Phenotypic Assays for Testing Fungal Susceptibility

### 2.1. Reference AFST Methods

Currently, phenotypic assays to perform in vitro AFST for either yeasts or filamentous fungi (also termed molds) include two universally recognized standard methods, CLSI [12,13] and EUCAST [14,15], which apply the broth microdilution method (BMD). Both measure antifungal activity, expressed as the minimum inhibitory concentration (MIC) of an antifungal drug, which indicates the minimal drug concentration that inhibits fungal growth. Despite some methodological differences (e.g., glucose concentration, inoculum size, reading endpoint, etc.) between the two [16], CLSI and EUCAST have been proven to yield, upon completion of testing, comparable MIC data for all classes of antifungal agents [19,20]. In particular, the EUCAST method uses a higher percentage of glucose (2%) in the test medium to facilitate increased fungal growth [14,15], which may be particularly advised when testing molds. To accelerate the time to AFST results, both methods include (at least for *Candida* species) a recommended incubation time of 24 h [20]. Based on different absolute MIC values generated, EUCAST and CLSI have established divergent CBPs (i.e., MIC thresholds used to classify isolates as susceptible or resistant) for *Candida* species [21,22]. Contrary to CLSI, which has not set CBPs against any molds (including *Aspergillus* species) [23], EUCAST provides mold CBPs, with species-related CBPs determined for *A. fumigatus*, *A. flavus*, *A. nidulans*, *A. niger*, and *A. terreus* [21]. The two standards propose the concept of minimum effective concentration (MEC) for reading echinocandin AFST results of molds. However, MEC determination is not always easy because of its reliance on assessing the transition point of hyphae from normal to aberrant forms, which often requires microscopic observation. In general, reference BMD assays are technically demanding and not intended for routine laboratory practice. Additionally, the interlaboratory variability in caspofungin MICs noted with *Candida* species may significantly hinder the use of both CLSI and EUCAST methods [24]. Nonetheless, they hold great value as indispensable comparators in evaluating performance studies of commercial methods [25,26], such as those discussed below.

In one large study published recently [27], good correlation was obtained between EUCAST (EDef 7.2) and CLSI (M27-A3) for amphotericin B, flucytosine, anidulafungin, caspofungin, micafungin, fluconazole, isavuconazole, itraconazole, posaconazole, and voriconazole among 357 isolates of *Candida* species, showing >93% categorical agreement for all antifungal agents tested. Low agreement mainly regarded testing of amphotericin B, anidulafungin, and isavuconazole against *C. glabrata*, and caspofungin against *C. parapsilosis*, *C. tropicalis*, and *C. krusei*, leading to further calls for more harmonization.

### 2.2. Commercial AFST Methods

As previously reviewed [16], either BMD methods, which use color endpoints due to metabolic dye (e.g., AlamarBlue) incorporated into growth media (e.g., Sensititre^TM^ YeastOne^TM^ (SYO; Thermo Fisher Scientific, Waltham, MA USA)), or agar-based methods, which use concentration gradients of antifungals that diffuse into growth media (e.g., Etest^®^; AB Biodisk, Solna, Sweden), are modifications of the CLSI/EUCAST reference methods. A valid alternative to these (manual) assays is to perform AFST through automated means (e.g., VITEK^®^ 2 system; bioMérieux, Marcy-l’Étoile, France). These phenotypic methods are per se limited by requiring a pure culture of the infecting organism before testing. Nevertheless, SYO, Etest, and VITEK 2 are commercial methods widely used for in vitro AFST of *Candida* and/or *Aspergillus* species, and are thought to be superior to reference methods in use, convenience, and flexibility [16].

Since its introduction in routine microbiology laboratories, the SYO microdilution antifungal panel—allowing simultaneous testing of amphotericin B, echinocandins, and triazoles—has been extensively evaluated for yeasts, becoming the focus of large hospital studies [28,29,30]. For CLSI CBPs/ECVs to assign susceptibility (or the WT phenotype) to systemically active antifungal agents (the SYO’s manufacturer recommends using CLSI CBPs), two recent studies reported on SYO MIC results for *Candida* species. In the study by Posteraro et al. [29], susceptibility/WT rates to amphotericin B and flucytosine were over 97% in all yeast isolates (*n* = 1250, including *Candida* and non-*Candida* species). Rates for fluconazole (excluding *C. krusei*), itraconazole, and voriconazole were 98.7% in *C. albicans*, 92.3% in the *C. parapsilosis* species complex, 96.1% in *C. tropicalis*, 92.5% in *C. glabrata*, and 100% in both *C. guilliermondii* and *C. krusei*. Rates for echinocandins were 99.7% to 99.8% in all *Candida* species. Similarly, Xiao et al. [30] found that over 99.3% of the isolates (*n* = 1072, including all common non-*albicans Candida* species) had a WT phenotype to amphotericin B and flucytosine. Susceptibility/WT rates for azoles among *C. parapsilosis* species complex isolates were ≥97.5%. Among ~14.3% of fluconazole-resistant *C. glabrata* isolates, 11.6% were cross-resistant to fluconazole and voriconazole. All *C. krusei* isolates were susceptible/WT to voriconazole, posaconazole, and itraconazole, whereas 97.7% to 100% of isolates were susceptible to caspofungin, micafungin, and anidulafungin.

Echinocandins represent first-line treatment of invasive *Candida* infections [5,31,32]. Acquired echinocandin resistance is mainly observed among *C. albicans* and *C. glabrata* and is associated with mutations in two narrow hot spot (HS) regions (HS1 and HS2) of *FKS1* (*C. albicans* and *C. glabrata*) and *FKS2* (*C. glabrata* only) genes [33]. Eschenauer et al. [28] underscored that adopting CLSI CBPs for caspofungin may overstate the rates at which isolates of *C. glabrata* and *C. krusei* are nonsusceptible to caspofungin. While clinical microbiology laboratories should use micafungin and anidulafungin as surrogate markers to predict susceptibility or resistance to caspofungin [34,35], some authors have tried to establish ECVs using the SYO method [36,37]. Espinell-Ingroff et al. [37] calculated SYO ECVs for echinocandins and eight *Candida* species. Notably, SYO ECVs for anidulafungin, caspofungin, and micafungin correctly classified as non-WT 88.9% (72/81), 91.4% (74/81), and 93.8% (76/81), respectively, of *Candida* isolates with known *FKS* mutations. Despite their sensitivity for identifying hot spot mutations, the positive and negative predictive values of these ECVs in routine clinical application were not determined. Very recently, Kritikos et al. [38] calculated echinocandin ECVs for *C. albicans* (*n* = 1277) and *C. glabrata* (*n* = 347) tested by SYO and assessed their ability to identify *FKS* mutants in a 10-year candidemia survey from the FUNGINOS network. Among 70 isolates with MIC ≥ ECV for any echinocandin and then sequenced, no *FKS* mutation was found in the 52 “limit WT” isolates (MIC = ECV for at least one echinocandin), suggesting an excellent negative predictive value of these ECVs. Among the 18 “non-WT” isolates (MIC > ECV for at least one echinocandin), *FKS* mutations were found in the only two isolates with MIC > ECV for all three echinocandins, but not in the isolates having a “non-WT” phenotype for only one or two echinocandins. However, validating these SYO EVCs in settings with higher rates of echinocandin resistance remains to be done.

Despite being a rarity, echinocandin non-WT clinical isolates of *Aspergillus* species with MECs above the ECV were identified [39]. Reduced susceptibility of *Aspergillus fumigatus* isolates to echinocandins in vitro can develop via *FKS*-dependent and *FKS*-independent mechanisms [40,41]. As previously mentioned, SYO is a colorimetric adaption of the CLSI BMD method, which is based on either the M27-A3 standard for yeasts or the M38-A2 standard for molds [12,13]. SYO color endpoints are determined visually as the AlamarBlue color changes from blue (0% growth) to red (100% growth), and only an intermediate purple color can be indicative of 50% growth. Until now, only one published study has evaluated the performance of SYO versus the reference CLSI BMD method for in vitro susceptibility testing of *Aspergillus* species to echinocandins. Siopi et al. [42] tested 39 isolates of *A. fumigatus*, *A. flavus*, and *A. terreus*, including two echinocandin-resistant *A. fumigatus* strains. Overall, the best agreement with the CLSI M38-A2 method was with an inoculum of 10^4^ CFU/mL, incubation times of 20 h for *A. flavus* and 30 h for *A. fumigatus* and *A. terreus*, and reading the first purple well. However, the agreement was poor for caspofungin (0 to 54%) but good for micafungin (77 to 100%), whereas it was inconclusive for anidulafungin because all *Aspergillus* isolates yielded off-scale SYO color endpoints. Interestingly, both resistant isolates converted AlamarBlue within 24 h at high concentrations, indicating that detection of echinocandin-resistant *Aspergillus* isolates may be feasible with the SYO method. Hence, the authors noticed that the current AlamarBlue concentration may be sufficient for the slower-growing *A. fumigatus* and *A. terreus* but not for the fast-growing *A. flavus*, highlighting the need for further exploration and validation of the optimal AlamarBlue concentration. By the authors’ opinion [42], in an average clinical laboratory, a good compromise could be inoculating *A. flavus* isolates in the afternoon and reading them the next morning, and inoculating *A. fumigatus* and *A. terreus* isolates in the morning and reading them the next afternoon. Therefore, the authors advised not considering 48 h of incubation for SYO and echinocandins, instead proposing susceptibility testing of *Aspergillus* species against amphotericin B, itraconazole, posaconazole, and voriconazole.

Consistent with the role of triazoles in the treatment and/or prophylaxis of invasive aspergillosis [43], CLSI-based ECVs for itraconazole, posaconazole, voriconazole, and, lately, isavuconazole were established for *Aspergillus* species (*A. fumigatus*, *A. flavus*, *A. terreus*, *A. niger*, *A. nidulans*, and *A. versicolor*) to aid in the early identification of clinical isolates with acquired resistance mechanisms [44,45]. Different patterns of azole resistance (i.e., multi-azole, pan-azole, or single-azole resistance) in *A. fumigatus* are, to various extents, associated with mutations in the coding region of the sterol 14α‒demethylase-encoding gene *CYP51A*, as well as tandem repeats of 34, 46, and 53 bp upstream in the promoter region of *CYP51A* [46]. The most frequently observed mutation is TR_34_/L98H, which consists of a 34 bp tandem repeat (TR_34_) in the gene promoter region combined with the substitution of leucine 98 for histidine (L98H) [47]. Mello et al. [48] comparatively evaluated the in vitro activity of posaconazole, voriconazole, and itraconazole against clinical isolates from common (*n* = 59) and uncommon (*n* = 27) *Aspergillus* species using the SYO and CLSI M38-A2 methods. The overall essential agreement between SYO MICs and CLSI MICs was 100% for voriconazole and 96.5% for both itraconazole and posaconazole. Calculating only for isolates of *A. fumigatus*, *A. flavus*, *A. terreus*, and *A. niger*, the essential agreement value was unchanged for voriconazole (100%), but increased for posaconazole (98.3%) and decreased for itraconazole (94.9%). When interpreting MICs according to CLSI ECVs for the 21 *A. fumigatus*, 19 *A. flavus*, 12 *A. terreus*, 7 *A. niger*, and 5 *A. nidulans*, the categorical agreement between the methods was 96.9% (62/64 isolates) for posaconazole, 98.4% (63/64 isolates) for voriconazole, and 93.7% (60/64 isolates) for itraconazole. Of 10 *A. fumigatus* isolates with *CYP51A* alterations, all but two isolates exhibited non-WT phenotypes (MIC > ECV) for posaconazole and voriconazole (or itraconazole) obtained with both the SYO and CLSI methods. Interestingly, one of the two isolates (both harboring the TR_34_/L98H mutation) had a posaconazole non-WT phenotype that was determined by the CLSI method but not by the SYO method. The other isolate had a voriconazole non-WT phenotype that was determined by the SYO method but not by the CLSI method. Posaconazole MICs ≤0.5 μg/mL provided separation between WT isolates of *A. fumigatus* and those harboring mutations in the *CYP51A* gene. Based on these findings, SYO and CLSI are equivalent methods for testing triazole susceptibility in *Aspergillus* species. It is worth noting that, in line with Siopi et al.’s study [42], Mello et al. [48] performed visual readings of SYO MICs regardless of color changes, which were necessary because of prolonged incubation times (>24 h) of the SYO panels.

Compared to SYO, gradient diffusion assays such as Etest/MIC Test Strips are easy to perform but expensive when used on a larger scale [46]. However, in the context of azole resistance, recent reports show the reliability of gradient MIC approaches that represent alternative means of quantitatively determining in vitro susceptibility to antifungal agents [49,50]. Arendrup et al. [49] evaluated the MIC Strip Isavuconazole test (Liofilchem, Roseto degli Abruzzi, Italy), the only commercially available isavuconazole susceptibility test, against EUCAST EDef 9.3 by using 40 WT and 39 *CYP51A* mutant isolates of *A. fumigatus*. MICs were determined by two independent readers and were interpreted according to the EUCAST isavuconazole epidemiological cutoff value (ECOFF; 2 μg/mL) and clinical breakpoint (1 μg/mL). Using the strip’s full inhibition endpoint, the essential agreements with the EUCAST reference method (at ±1 and ±2 twofold dilutions) were 73–75% and 89–90% [49], which were better than previously found for the isavuconazole Etest (no longer available) when compared with the CLSI reference method [51]. The categorical agreement was >91%, with 6.3–8.9% very major errors (defined as isolate categorized as resistant by the EUCAST reference method but susceptible by the strip test) and 0–1.3% major errors (defined as isolate categorized as susceptible by the EUCAST reference method but resistant by the strip test). Interestingly, very major errors included four isolates with a WT *CYP51A* genotype that either may have been harboring other resistance mechanisms or may have been isolates misclassified as resistant by the EUCAST reference method because of the restrictive clinical breakpoint [52]. By the authors’ opinion [49], interpreting the MICs obtained with commercial tests by such a breakpoint may generate a higher risk of misclassification unless the susceptibility test is very well standardized against the AFST reference method. Finally, the discrimination between WT and TR_34_/L98H mutant isolates was greater for the MIC strip test, making it potentially suitable for detecting resistant environmental mutants, provided the full inhibition endpoint is used [49]. Despite being promising, the performance of the MIC Strip Isavuconazole test needs to be confirmed in a multicenter study. More recently, Idelevich et al. [50] evaluated Etest (bioMérieux) and MIC Test Strip (Liofilchem) for itraconazole, posaconazole, and voriconazole, and only MIC Test Strip for isavuconazole, against the EUCAST reference method by using clinical consecutive isolates (*n* = 24) and control strains (*n* = 15, with defined resistant or susceptible phenotypes and genotypes) of *A. fumigatus*. Regarding the clinical isolate collection, both assays had good performance for itraconazole and voriconazole. However, the MIC Test Strip showed low agreement with the EUCAST reference method due to a high rate of minor errors (defined as false categorization involving intermediate results) and major errors. While MIC Test Strip performed well for isavuconazole, all clinical isolates determined to be resistant to a particular azole by the EUCAST reference method were correctly detected as resistant by both gradient diffusion assays. Regarding the control strain collection, both gradient diffusion tests had worse performance. In particular, three major errors occurred with MIC Test Strip for isavuconazole (one strain with M220V *CYP51A* allele and two strains with WT *CYP51A* alleles). The authors [50] concluded that both assays can reasonably be used for azole susceptibility testing with *A. fumigatus*.

Concerning the echinocandin susceptibility testing of *Candida* species with Etest (bioMérieux), Bougnoux et al. [53] recently enrolled 16 French hospital centers to obtain 933 *Candida* isolates that were tested against micafungin. MICs were also determined by the EUCAST reference method at a single center. The overall essential agreement between EUCAST and Etest results was high (98.5% at ±2 twofold dilutions and 90.2% at ±1 twofold dilutions), whereas a categorical agreement of 98.2% was observed for the 742 isolates belonging to the five species for which clinical breakpoints or ECOFFs were available. The authors demonstrated that the Etest gave micafungin susceptibility results that were very similar to those given by the EUCAST reference method under routine laboratory testing. Meanwhile, in vitro micafungin resistance rates for the *Candida* species mainly isolated from clinical samples were low (<2% for *C. albicans* and *C. parapsilosis* and 3.9% for *C. glabrata*). More recently, a 1-year survey conducted on 104 *Candida* species isolated from blood cultures in an Austrian hospital by Aigner et al. [54] showed that the levels of essential agreement with the EUCAST reference method were 97% and 92% for anidulafungin and micafungin. The categorical agreement of the Etest was 99% for both anidulafungin and micafungin, irrespective of the clinical breakpoints applied (EUCAST versus CLSI). Only one *C. glabrata* isolate was classified as echinocandin-resistant by Etest. Unfortunately, the lack of resistant isolates in this study’s collection hindered making any recommendations regarding accurate resistance detection by the Etest method.

## 3. Nonconventional Phenotypic Assays for Testing Fungal Susceptibility

### MALDI-TOF Mass Spectrometry-Based AFST Methods

In many European clinical microbiology laboratories such as ours, the advent of matrix-assisted laser desorption ionization time-of-flight mass spectrometry (MALDI-TOF MS) has drastically altered the routine diagnostic workflow [55,56]. As a result, MALDI-TOF MS offers the chance to identify almost all microbial genera and species with unprecedented reliability, rapidity, and cost-effectiveness [57]. However, this has occurred quickly for bacteria [58] but not so quickly for fungi [59]. Difficulties linked to the complexity of the fungal cell hampered early optimization of sample analytical procedures and, consequently, large-scale adoption of MALDI-TOF MS in medical mycology [59]. To date, MALDI-TOF MS analysis is suited for microbial isolates cultured from primary samples [60] or positive blood cultures [61], significantly reducing the turnaround time compared to biochemical [62] or nucleic acid–based techniques, such as DNA sequencing [63]. However, high MALDI-TOF MS performance, particularly for cryptic species within the *Candida* and *Aspergillus* species complexes, can only be achieved with the appropriate databases provided by some marketed MALDI-TOF MS systems [59,60]. While susceptibility testing methods are not directly applicable to primary samples [64], some recent studies have reported success with phenotype-centered (or semimolecular) MALDI-TOF MS methods for AFST [65].

In keeping with previous work that introduced the minimal profile change concentration (MPCC) as a new endpoint for AFST [66], our research group developed MALDI-TOF MS–based assays for testing the echinocandin susceptibility of fungal species. In one of the first assays, De Carolis et al. [67] obtained mass spectra from fungal cells exposed to different caspofungin concentrations for 15 h, and then matched the “intermediate” mass spectra with each of the “extreme” mass spectra using composite correlation index (CCI) analysis. MPCC represents the CCI value at which a spectrum is more similar to the spectrum observed at the maximal caspofungin concentration (maximum CCI) than the spectrum observed at the null caspofungin concentration (null CCI). The authors showed that MPCC values approximated MIC (or MEC) values for 100% of *Candida* and *Aspergillus* isolates tested (in total, 44 among WT and *FKS1* mutant isolates). In the second assay, which was the first one simplified (here named MS-AFST), Vella et al. [68] provided discrimination between susceptible and resistant isolates of *C. albicans* after 3 h of exposure of fungal cells at three antifungal drug levels: no drug (null concentration), intermediate (“breakpoint”), and maximum (maximal concentration). By means of this “three-point” assay, isolates were susceptible or resistant when the CCI values obtained by matching their breakpoint spectrum with their maximum spectrum were, respectively, higher or lower than the CCI values obtained by matching their breakpoint spectrum with their spectrum at null concentration. Using this criterion, 100% (51/51) and 90.9% (10/11) of the isolates tested yielded MALDI AFST results that were in accordance with the WT or *FKS1*-mutant genotype, respectively.

To extend our findings, Vella et al. [69] tried to validate the 3 h MS-AFST assay with a panel of 80 clinical isolates of *C. glabrata* tested against echinocandin (anidulafungin) and triazole (fluconazole) antifungal agents. Although acquired azole resistance in *Candida* species is multifaceted [70], the induction of drug efflux encoded by *CDR* genes and regulation of their expression by mutations in the transcription factor CgPdr1 (encoded by the *CgPDR1* gene) represent the most clinically relevant molecular mechanisms [71,72]. The study [69] revealed that 85.0% (68/80) and 96.2% (77/80) of isolates had classification results for anidulafungin and fluconazole that fully agreed with those obtained by the *FKS1*/*FKS2* genotype or *CgCDR1*/*CDR2* overexpression antifungal-resistance mechanisms. When analyzing the MS-AFST results according to the *FKS1*/*FKS2* genotype, agreement was 100% (6/6) for isolates with a mutated *FKS1* gene and 25.0% (4/16) for isolates with a mutated *FKS2* gene. This resulted in 15.0% of incorrect classifications for anidulafungin that involved *FKS2* HS1 mutations. According to the CLSI reference method, MS-AFST assays yielded as many as 11 very major errors (i.e., a resistant isolate misclassified as susceptible) with anidulafungin, and only two very major errors with fluconazole. Interestingly, discrepancies could be resolved with MS-AFST assays performed at 15 h of exposure to both antifungal drugs; in this case, MPCC values were coincident with the MICs for those isolates showing discrepant results. Taken together, these findings demonstrate that MS-AFST in the 3 h format failed to detect *C. glabrata* isolates with echinocandin-associated *FKS2* mutations.

Contemporarily, but independently from us, Gitman et al. [73] explored the MALDI-TOF MS–based method to differentiate WT (no acquired resistance) from non-WT (acquired resistance) isolates of 20 *Aspergillus* species (including 17 *A. fumigatus*) with respect to voriconazole (MIC > ECV; 1 μg/mL). Four of 17 *A. fumigatus* were phenotypically and genotypically resistant to voriconazole, whereas two isolates of *Aspergillus ustus* and one isolate of *Aspergillus calidoustus* served as controls due to their intrinsic low azole susceptibility. Except for two of four mutant isolates misclassified as WT, MALDI-TOF MS MPCCs at the 24 h time point were within ±1 dilution of the BMD MICs (determined by the SYO method) for all *Aspergillus* isolates. In line with our studies [67,68,69], slightly longer incubation times (30 and 48 h) allowed for accurate detection of isolates as WT or non-WT by MS-AFST, in complete agreement with the *CYP51A* gene sequence analysis results. Remarkably, using the “three-point” assay (no drug, 16 μg/mL voriconazole, and voriconazole ECV), the authors [73] obtained the same findings, requiring 30 to 48 h of incubation with the drug prior to MALDI-TOF MS analysis. It is likely that some fungal isolates requiring additional time relates to the impossibility of their reaching enough growth and, consequently, level of expressed proteins to allow for correct classification by the MS-AFST assay [69,73].

In the above-mentioned study, Vella et al. [69] obtained reproducibility rates of 98.7% and 97.5% when testing *C. glabrata* against anidulafungin and fluconazole, respectively, with only three inconsistencies, subsequently arbitrated by a third run before including them in the MS-AFST analysis. In one study performed similarly to that described originally by us [67], Saracli et al. [74] showed that the reproducibility of the MALDI-TOF MS–based assay for discriminating susceptible and resistant isolates of *Candida* species (35 *C. albicans*, 35 *C. glabrata*, and 37 *C. tropicalis*) to triazoles varied between 54.3% and 82.9%. However, the reproducibility was higher for *C. glabrata* isolates (77.1% for fluconazole) than for isolates from other *Candida* species. In addition, applying a 5% tolerance for evaluation of the CCI ratio (CCI_max_/CCI_null_ ratio >1 (MS-AFST assay-classified as susceptible); CCI_max_/CCI_null_ ratio <1 (MS-AFST assay-classified as resistant)) did result in a decrease in the percentages of very major and major errors by up to 33.3%.

## 4. How to Better Use Phenotypic Fungal Susceptibility Results in the Clinic Setting

Intended to reliably identify patients whose infection is likely to respond to a given antifungal agent, in vitro susceptibility testing has improved our ability to predict the outcome of therapy [9], yet is constantly confronted with increasing resistance to antifungal agents [3]. Since the widespread use of triazoles in early 1990, antifungal resistance in both *Candida* and *Aspergillus* has become a serious public health problem [75,76]. The rise in echinocandin resistance, azole resistance, and cross-resistance to two or more antifungal classes (multidrug resistance) in pathogenic fungi [77] has involved species of *Candida* such as *C. glabrata* [78] and, lately, *C. auris* [79]. Additionally, the incidence of azole-resistant *A. fumigatus* has jeopardized outcomes for high-risk patients, because the exclusion of azole antifungal drugs from prophylaxis or first-line treatment of invasive aspergillosis would limit drug choices [43]. However, we are conscious that various host, drug, and fungal factors contribute to therapeutic failures, and there is no absolute association between in vitro MIC and clinical response [75]. This has precluded CLSI or EUCAST from establishing CBPs for some antifungal agents and fungal species, even though both the CLSI and EUCAST methods, which result in different CBPs, are grounded in pharmacodynamic responses in animal models and patients [75].

**Figure 1:**
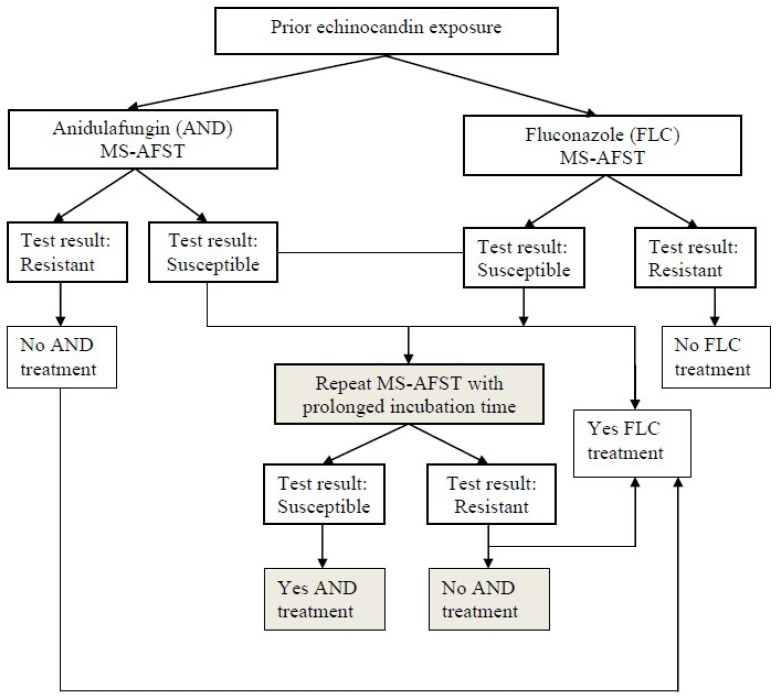
Potential treatment of invasive *C. glabrata* infection based on mass spectrometry– antifungal susceptibility testing (MS-AFST) results. In a clinical context of prior echinocandin exposure, results of susceptibility or resistance to anidulafungin and/or fluconazole within 3 h or, in cases of isolates with *FKS2* HS1 mutations, 6–12 h after testing may guide the appropriate administration of antifungal therapy [69].

Concerning *Candida*, antifungal susceptibility is predictable if the infecting organism is identified to the species level, but individual isolates may not follow this course, thus requiring antifungal testing [9]. Importantly, the Infectious Diseases Society of America (IDSA) guidelines for the management of candidiasis recommend routinely performing AFST for *C. glabrata* against azoles and echinocandins [5]. The same guidelines mention that routine testing for *Candida* species other than *C. glabrata* has less value [5]. Nevertheless, we agree with the opinion by Ostrosky-Zeichner and Andes [9] that routinely testing antifungal susceptibility of all bloodstream and sterile site isolates of *Candida* species may be helpful to provide an index for susceptibility trends and the emergence of resistance locally and regionally. However, in a resource-restricted environment, AFST should focus on isolates from cases of treatment failure, breakthrough infection, or limited therapeutic options, which are consequences of underlying comorbidities, adverse events, or previous antifungal use [9]. Consistent with this, in a scenario of prior echinocandin exposure, Vella et al. [69] proposed that rapid detection of *C. glabrata* isolates as fluconazole-resistant by the MS-AFST assay could alert clinicians to the potential presence of anidulafungin resistance in these isolates (Figure 1). Despite representing a personal view of the utility of this approach (and not a recommendation), Figure 1 underlines that using anidulafungin as a surrogate marker would lead to a scenario in which, if no resistance is detected, either of the three echinocandins (not only anidulafungin) could be administered.

**Figure 2:**
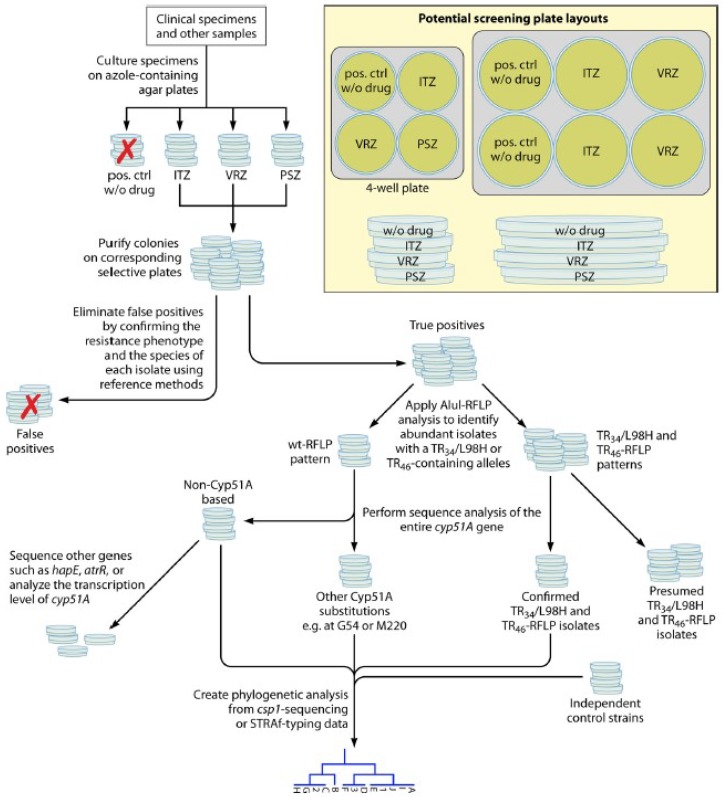
Potential screening of azole-resistant *A. fumigatus* isolates. Shown is a stepwise workflow that eliminates false-positive results (indicated by a red X) and can be helpful to build up local epidemiological data on the prevalence and phylogenetic linkage of resistant isolates. In this figure, true positives are derived from the growth of isolates on selective agar plates containing drug concentrations that range from 0.5 μg/mL for posaconazole (PSZ) to 1 to 4 μg/mL for itraconazole (ITZ) or voriconazole (VRZ). Then, isolates are identified at the species level by multilocus DNA sequencing [46] (got permission to use).

Concerning *Aspergillus*, the IDSA guidelines for the management of aspergillosis recommend performing susceptibility testing against azoles primarily for patients who fail to respond to therapy or for epidemiological purposes [6]. However, these guidelines do not specify laboratory-testing procedures for the isolation of *Aspergillus* from respiratory tract samples [46]. Recently, an international expert panel convened to deliberate the management of azole-resistant invasive aspergillosis, concluding that in culture-positive cases, in vitro susceptibility testing is highly indicated when antifungal therapy is intended [80]. Up to five colonies need to be tested in patients who are to receive antifungal therapy in geographic regions with azole resistance [80], which is also recommended by the European guidelines for aspergillosis published in early 2018 [81]. Clinically used triazole antifungals are derivatives of either fluconazole (voriconazole and isavuconazole) or ketoconazole (itraconazole and posaconazole) as the lead compound. This correlates with the cross-resistance phenotypes observed in clinical (and environmental) *A. fumigatus* isolates with the *CYP51A* mutations that are within the itraconazole/voriconazole and voriconazole/isavuconazole compound pairs [46]. Therefore, while at least voriconazole and itraconazole are recommended as screening drugs, the VIPcheck (Nijmegen, The Netherlands), a commercial agar-based method that consists of four wells containing voriconazole, itraconazole, posaconazole, or a growth control, was developed for easily discriminating between azole-susceptible and -resistant isolates of *A. fumigatus* [82]. Clinical screening studies are encouraged to generate epidemiological data, which in turn may help to reassess clinical treatment options on a local or national basis. In this context, Dudakova et al. [46] proposed a workflow for evaluating *A. fumigatus* isolates from such screening studies that identifies true positives and yields robust data on the prevalence and phylogenetic relatedness of resistant isolates (Figure 2). In the authors’ own experience, the species of each isolate can be readily determined via MALDI-TOF MS using the commercial databases. The isolates with a dark blue-green appearance, which is common to *A. fumigatus* and its sibling species, but not a reliable MALDI-TOF MS identification as *A. fumigatus*, prove to be cryptic species not yet included in the scheme presented (Figure 2). Additionally, the authors compiled the MIC values correlating with individual amino acid substitutions in the *CYP51A*-encoded enzyme for interpretation of DNA sequencing data, especially in the absence of cultured *A. fumigatus* isolates.

AFST continues to be instrumental in identifying cases of invasive fungal infections associated with elevated MICs and in detecting antifungal resistance phenotypes, i.e., determining which antifungal agents are likely to be clinically inactive [83]. However, for patients with candidemia who are experiencing clinical response and are infected by seemingly resistant isolates, there is the possibility of MIC misinterpretation due to artifacts such as the paradoxical growth seen with echinocandins and the trailing effect seen with azoles [84]. On the other hand, the clinical microbiology laboratory is often unable to provide information in a period that can inform initial antifungal treatment decisions. For example, the total turnaround time for the current antifungal resistance detection process for patients with bloodstream infections is 3–5 days, since it requires three overnight growth steps (blood culture, subculture, and AFST culture) [63]. Molecular detection of resistance genes, especially when applied to primary clinical samples [85,86], may be a useful adjunct for fungal surveillance and disease diagnosis [46]. However, these methods generally do not provide a “comprehensive assessment of what an organism can be treated with” [87]. Nowadays, growth-based phenotypic AFST is still required to fully determine the susceptibility profile of the infecting pathogen. Therefore, recent efforts have led to the development of innovative technologies that promise to shorten the turnaround time for phenotypic AFST results [88]. Designed to identify common causes of bacteremia (~90 min) as well as perform antimicrobial susceptibility testing (~7 h) using positive blood cultures, the Food and Drug Administration (FDA)-cleared Accelerate PhenoTest^TM^ Blood Culture kit represents an important breakthrough in the development of rapid phenotypic antimicrobial susceptibility testing [89]. Further application of this or other game-changing technologies such as MALDI-TOF MS [90] to fungi would allow for similar gains in AFST performance, along with improvements of fungal diagnostic capability [91].

## 5. Conclusions

As for *Candida* and *Aspergillus*, the infection-related mortality rates are still unacceptably high, despite recent advances in prophylaxis, early diagnosis, and treatment of fungal diseases [1]. Antifungal prophylaxis with antimold-active azole compounds (posaconazole or voriconazole) to reduce the incidence of invasive mold infections in high-risk patients may be associated with breakthrough infections caused by rare multidrug-resistant molds [92]. Therefore, accurate determination of antifungal susceptibility of fungi, which may also include non-*Aspergillus* molds (*Mucorales*, *Fusarium* spp., or *Scedosporium apiospermum* complex), is mandatory at least in specific situations during the care of patients with invasive fungal infections [9]. Whether the conventional antimicrobial susceptibility tests are still useful will depend on how fast the march toward rapid phenotypic antimicrobial susceptibility testing is [87]. More work is expected to extend MALDI-TOF MS–based AFST to clinically relevant fungal pathogens other than *Candida* or *Aspergillus*, because there are no conceptual hurdles to do this [90]. In the future, the success or failure of newly emerged technologies, as measures of improved patient outcomes, will depend primarily on how great the local prevalence of antifungal resistance is and how rationally the technology is integrated into the clinical microbiology laboratory practice.
